# Effect of Cerium Oxide on Kidney and Lung Tissue in Rats with Testicular Torsion/Detorsion

**DOI:** 10.1155/2022/3176455

**Published:** 2022-03-22

**Authors:** Aycan Ozdemirkan, Ali Can Kurtipek, Aysegül Kucuk, Cagri Ozdemir, Suleyman Yesil, Saban Cem Sezen, Mustafa Kavutcu, Mustafa Arslan

**Affiliations:** ^1^Faculty of Medicine, Department of Anesthesiology and Reanimation, Gazi University, Ankara, Turkey; ^2^Department of Internal Medicine, Ankara City Hospital Health Sciences University, Ankara, Turkey; ^3^Faculty of Medicine, Department of Physiology, Kütahya Health Sciences University, Kütahya, Turkey; ^4^Faculty of Medicine, Department of Urology, Gazi University, Ankara, Turkey; ^5^Faculty of Medicine, Department of Histology and Embryology, Kırıkkale University, Kırıkkale, Turkey; ^6^Faculty of Medicine, Department of Medical Biochemistry, Gazi University, Ankara, Turkey; ^7^Life Sciences Application and Research Center, Gazi University, Ankara, Turkey

## Abstract

**Introduction:**

Testicular torsion is a surgical emergency that results in testicular ischemia as a result of rotation of the spermatic cord around itself. Oxidative damage occurs in the testis and distant organs with the overproduction of free radicals and overexpression of proinflammatory cytokines by reperfusion after surgery. In this study, we aimed to investigate the effects of cerium oxide (CeO_2_), an antioxidant nanoparticle, on lung and kidney tissues in testicular torsion/detorsion (T/D) in rats.

**Materials and Methods:**

After ethics committee approval, 24 rats were equally (randomly) divided into 4 groups. Left inguinoscrotal incision was performed in the control (C) group. In group CeO_2_, 0.5 mg/kg CeO_2_ was given intraperitoneally 30 minutes before inguinoscrotal incision. In group T/D, unilateral testicular T/D was achieved by performing an inguinoscrotal incision and rotating the left testis 720° clockwise, remaining ischemic for 120 minutes, followed by 120 minutes of reperfusion. In group CeO_2_-T/D, 0.5 mg/kg CeO_2_ was given intraperitoneally 30 minutes before testicular T/D. At the end of the experiment, lung and kidney tissues were removed for histopathological and biochemical examinations.

**Results:**

Glomerular vacuolization (GV), tubular dilatation (TD), tubular cell degeneration and necrosis (TCDN), leukocyte infiltration (LI), and tubular cell spillage (TCS) in renal tissue were significantly different between groups (*p* = 0.012, *p* = 0.049, *p* < 0.003, *p* = 0.046, and *p* = 0.049, respectively). GV and TCDN were significantly decreased in group CeO_2_-T/D compared to group T/D (*p* = 0.042 and *p* = 0.029, respectively). Lung tissue neutrophil infiltration, alveolar thickening, and total lung injury score (TLIS) were significantly different between groups (*p* = 0.006, *p* = 0.001, and *p* = 0.002, respectively). Neutrophil infiltration and TLIS were significantly decreased in group CeO_2_-T/D compared to group T/D (*p* = 0.013 and *p* = 0.033, respectively). Lung and kidney tissue oxidative stress parameters were significantly different between groups (*p* < 0.05). Renal tissue glutathione-s-transferase (GST), catalase (CAT), and paraoxonase (PON) activities were significantly higher, and malondialdehyde (MDA) levels were significantly lower in group CeO_2_-T/D than in group T/D (*p* = 0.049, *p* = 0.012, *p* < 0.001, and *p* = 0.004, respectively). GST and PON activities were higher, and MDA levels were lower in group CeO_2_-T/D than in group T/D in the lung tissue (*p* = 0.002, *p* < 0.001, and *p* = 0.008, respectively). *Discussion*. In our study, cerium oxide was shown to reduce histopathological and oxidative damage in the lung and kidney tissue in a rat testis torsion/detorsion model.

## 1. Introduction

Testicular torsion is a surgical emergency that accounts for 26% of acute testicular pain causes [1]. Testicular torsion results in ischemia by interruption of venous and arterial blood flow in the scrotum as a result of the rotation of the spermatic cord around itself. The incidence of testicular torsion peaks in men aged 12 and 18 years but can be seen in any age group [2]. Its incidence has been reported as 3.5 per 100000. Testicular torsion is mostly idiopathic and can occur with trauma in 20% of cases [3]. In testicular torsion, damage to the testis may occur depending on the degree and duration of rotation [4, 5]. When surgical detorsion in the treatment of testicular torsion is performed within 4-6 hours, the testis can be saved in the vast majority of cases [2]. However, with the detorsion, reperfusion will occur in the ischemic testicular tissue. At the end of the ischemia-reperfusion process, free radical production will increase.

Overproduction of free radicals causes irreversible DNA damage, changes in enzyme activities, damage to proteins, and many damages in the organism, such as the formation of new immunological structures [6]. It has also been shown to cause germ cell apoptosis [1]. Free radicals cause lipid peroxidation, especially by acting on fatty acids in the cell membrane. Lipid peroxidation is a very harmful chemical chain reaction that alters the membrane lipid structure and indirectly produces reactive aldehydes and damages the structure and functions of other cell components. Once this reaction starts autocatalytically, it continues as a chain and if not prevented, it destroys the cell membrane, breaks down the organelles, and causes the release of lysosomal enzymes and autolysis [6]. For this reason, testicular detorsion should be considered as a typical ischemia-reperfusion (I/R) injury. In addition, reactive oxygen radicals and neutrophil infiltration that occur during reperfusion play a role in the pathogenesis of distant organ injuries [7]. There are several agents used experimentally to reduce I/R injury after testicular torsion/detorsion (T/D) such as taurine [8–11], modafinil [12], sildenafil [13], and glutathione [14]. These treatments are aimed at eliminating or reducing the oxidative damage that occurs in I/R injury in the testis.

Many pathologies with associated oxidative damage respond to compounds that can scavenge reactive oxygen species (ROS). One of these compounds is cerium oxide (CeO_2_), an antioxidant nanoparticle that mimics the activities of catalase and superoxide dismutase enzymes and has scavenging properties of radical oxygen species; its efficacy has been demonstrated experimentally in many pathologies with oxidative damage [15–21]. Based on the findings of experimental studies, it is known that it reduces oxidative damage in I/R models and plays a protective role in distant organ damage [22–24]. Although there is little information in the literature about the effects of cerium on testicular tissue, it is not known whether it has a protective effect on distant organs in testicular ischemia-reperfusion injury [25–27]. The aim of this study is to show the effects of cerium oxide on kidney and lung tissues in testicular T/D in rats.

## 2. Materials and Methods

### 2.1. Animals and Experimental Protocol

This study was approved by the Gazi University Ethics Committee (Ethics number: G.U.ET-21-060). All experiments were performed according to standards of Guide for the Care and Use of Laboratory Animals in the Gazi University Animal Laboratory. A total of 24 male Wistar albino rats (4 rats in each cage) weighing between 250 and 300 grams were used. Rats were kept in a temperature-controlled (21 ± 1°C) and humidity-controlled (45–55%) room, which was maintained on a 12/12 reversed light cycle. Animals were fed with a standard pellet and allowed to drink water ad libitum. Animals were equally (randomly) divided into four equal groups (*n* = 6 each): (1) control (C), (2) cerium oxide (CeO_2_), (3) torsion/detorsion (T/D), and (4) cerium oxide-T/D (CeO_2_-T/D). Initially, all rats were anesthetized with 50 mg/kg intraperitoneal (ip) ketamine hydrochloride (VetaKetam, Vetagro) and ip 10 mg/kg xylazine hydrochloride (Rompun %2, BAYER).

Surgical procedures for testis torsion/detorsion are as follows: during the surgical procedures, rats were placed on a heating pad in order to maintain the body temperature. After the skin was shaved and washed with an antiseptic solution, left inguinoscrotal incision was performed using a sterile scalpel and forceps. Following exposing the left testis, unilateral testicular torsion was created by rotating the left testis 720° in a clockwise direction and fixing it within the hemiscrotum using a 4/0 atraumatic silk suture. Ischemia was observed with a dark purple color that appeared in the testicular tissue. Then, the skin was closed with 5/0 silk sutures and the testis was kept in torsion for 120 minutes. After 120 minutes, the incision was reopened by removing the sutures. The spermatic cord was detorsed; the testis was fixed within the hemiscrotum using a 4/0 atraumatic silk suture. Reperfusion was observed with the reappearance of pinkness in the testicular tissue. Heparin sodium (500 IU/kg, Nevparin, Mustafa Nevzat) was administered through the tail vein for the maintenance of reperfusion after occlusion. Then, the skin was closed with 5/0 silk sutures. The reperfusion phase was maintained for 120 minutes.

1. Control group (C): rats were only subjected to left inguinoscrotal incision
2. Cerium oxide group (CeO_2_): a left inguinoscrotal incision was performed. Cerium oxide was given (0.5 mg/kg, ip) 30 minutes before the incision. Cerium oxide was given at the predetermined dose [16]
3. Torsion-detorsion group (T/D): testis torsion/detorsion was performed surgically as described
4. Cerium oxide-torsion-detorsion group (CeO_2_-T/D): testis torsion/detorsion was performed surgically as described. Cerium oxide was given (0.5 mg/kg^,^ ip) 30 minutes before the ischemia period

CeO_2_ aqueous nanoparticle dispersion (<5 nm particle size, 20 wt% in H_2_O, 99.5% trace metals) was obtained from Sigma-Aldrich® (St Louis, MO, USA).

At the end of the experiments, rats were sacrificed under anesthesia. The lung and kidney tissues were excised for biochemical and histopathological analysis.

### 2.2. Histopathological Analysis

Lung tissue samples were removed and fixed in 10% neutral formalin solution. Then, the lungs were examined with light microscopy by the same pathologist, who was blinded to the study. A total of 10 random areas were evaluated with 200–400 times magnified microscopy in hematoxylin and eosin- (H&E-) stained sections. Stained slides were examined under a light microscope. Neutrophil infiltration and alveolar thickness are measured in each specimen for exposing the degree of lung injury area. Each parameter was scored as any (0 point), only a little (1 point), medium amount (2 points), or severe (3 points). The two scores were added and noted as total lung injury score (TLIS).

After routine fixation process, kidney specimens were embedded in paraffin blocks; then, tissue sections of 5 *μ* were mounted on slides for staining with hematoxylin and eosin (H&E). Histopathological evaluation under light microscopy was performed, and findings were scored using a scoring system by Bostan et al. [28]. Glomerular vacuolization (GV), tubular dilatation (TD), vascular vacuolization and hypertrophy (VVH), tubular cell degeneration and necrosis (TCDN), Bowman space dilatation (BSD), tubular hyaline cylinder (THC), leucocyte infiltration (LI), and tubular cell spillage (TCS) were scored using a scoring system: 0: no change; +1: minimal change; +2: medium; and +3: severe.

### 2.3. Biochemical Analysis

The lung and kidney tissues were first washed with cold NaCl solution (0.154 M) to discard blood contamination and then homogenized in Diax 900 (Heidolph Instruments GmbH & Co KG, Schwabach, Germany) at 1000 U for about 3 min. After centrifugation at 10,000 × g for about 60 min, the upper clear layer was taken.

For the measurement of malondialdehyde (MDA) levels, thiobarbituric acid (TBA) reactive substances assay was performed by the method described by Van Ye et al. [29]. The reaction with TBA at 80-90°C was used to determine the MDA level, as MDA or similar substances react with TBA and produce a pink pigment that has an absorption of maximum 532 nm. To ensure protein precipitation, the sample in room temperature was mixed with cold 20% (*w*/*v*) trichloroacetic acid and the precipitate was then centrifuged for 10 min at 3000 rpm at room temperature to form a pellet. An aliquot of the supernatant was then placed into an equal volume of 0.6% (*w*/*v*) TBA in a boiling water bath for 30 min. Following cooling, sample and blank absorbance was read at 532 nm and the results were expressed as nanomole per milligram of protein, based on a graph where 1,1,3,3-tetramethoxypropane had been used as our MDA standard.

The catalase (CAT) activity is based on the measurement of absorbance decrease due to H_2_O_2_ consumption at 240 nm as described by the Aebi H method [30].

Glutathione-s-transferase (GST) enzyme activity was measured using the method described by Habig et al. [31]. The GST activity method is based on the measurement of absorbance increase at 340 nm due to the reduction of dinitrophenyl glutathione (DNPG). The results were expressed in international unit per milligram of protein.

Paraoxonase- (PON-) 1 activity was measured as the rate of hydrolysis of paraoxon by monitoring the increase of absorbance at 405 nm and at 25°C described by Eckerson et al.'s method [32].

### 2.4. Statistical Analysis

Statistical Package for the Social Sciences (SPSS, Chicago, IL, USA) 20.0 for Windows was used. Each categorical variable was analyzed by the Kolmogorov-Smirnov test. Biochemical and histopathological parameters were tested by using the Kruskal-Wallis test, Bonferroni correction test, and Mann–Whitney *U* test. A statistical value of less than 0.05 was considered significant. All values were expressed as mean ± standard error (mean ± SE).

## 3. Results

**Figure 1:**
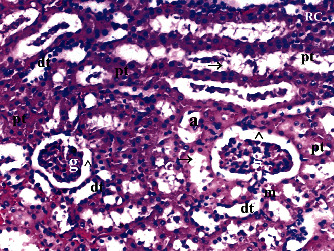
Renal tissue in the control group. RC: renal cortex; pt: proximal tubule; dt: distal tubule; g: glomerular; arrowhead: bowman space; arrow: dilated tubule; a: artery (H&E ×100).

**Figure 2:**
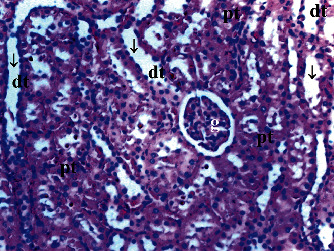
Renal tissue in the cerium oxide group. pt: proximal tubule; dt: distal tubule; g: glomerular; arrow: dilated tubule (H&E ×100).

**Figure 3:**
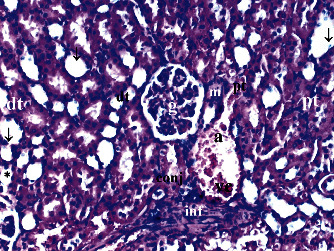
Renal tissue in the testicular torsion/detorsion group. pt: proximal tubule; dt: distal tubule; vc: vascular congestion; conj: capillary congestion; g: glomerulus; arrow: dilated tubule; m: macula densa; a: artery; ∗: degenerated glomerulus; inf: inflammation (H&E ×100).

**Figure 4:**
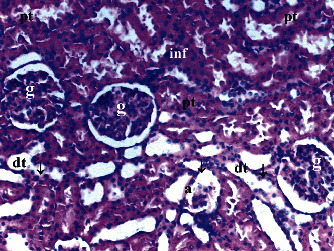
Renal tissue in the testis torsion/detorsion treated with cerium oxide group. pt: proximal tubule; dt: distal tubule; g: glomerulus; a: artery; arrow: dilated tubule; double arrow: macula densa; inf: inflammation (H&E ×100).

GV, TD, TCDN, LI, and TCS in the renal tissue were significantly different between groups (*p* = 0.012, *p* = 0.049, *p* < 0.003, *p* = 0.046, and *p* = 0.049, respectively). VVH, BSD, and THC were similar among groups (*p* = 0.107, *p* = 0.434, and *p* = 0.053, respectively). All renal histopathological findings, except VVH, BSD, and THC, were higher in group T/D compared to groups C and CeO_2_ (*p* > 0.05). GV and TCDN were significantly decreased in group CeO_2_-T/D compared to group T/D (*p* = 0.042 and *p* = 0.029, respectively) (Table 1, Figures 1–4).

**Figure 5:**
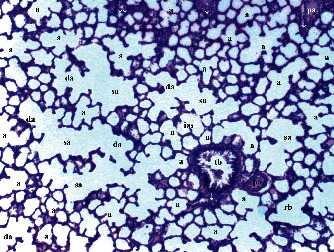
Normal-structural lung tissue parenchyma in the control group. a: alveolus; sa: saccus alveolaris; da: ductus alveolaris; tb: terminal bronchiole; pa: pulmonary artery; ↓: pulmonary vascular thickening (H&E ×40).

**Figure 6:**
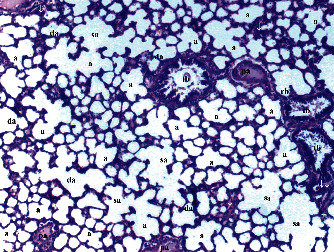
Mild neutrophilic infiltration and increased alveolar wall thickness in the cerium oxide group. a: alveolus; pa: pulmonary artery; rb: respiratory bronchiole; sa: saccus alveolaris; ↓↓: (septum) thickening (H&E ×40).

**Figure 7:**
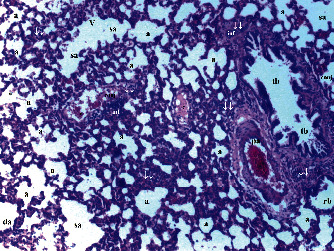
Severe neutrophilic infiltration and increased alveolar wall thickness in the testis torsion/detorsion group. a: alveolus; rb: respiratory bronchiole; inf: inflammation; ↓: pulmonary vascular thickening; ↓↓: (septum) thickening; conj: capillary congestion; vc: vascular congestion (H&E ×40).

**Figure 8:**
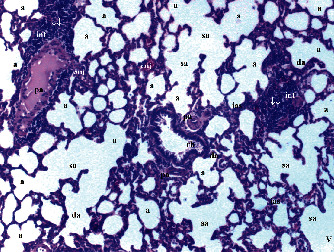
Mild neutrophilic infiltration and increased alveolar wall thickness in the testis torsion/detorsion treated with cerium oxide group. a: alveolus; da: ductus alveolaris; pa: pulmonary artery; rb: respiratory bronchiole; inf: inflammation; ↓: (septum) thickening; conj: capillary congestion; vc: vascular congestion (H&E ×40).

According to neutrophil infiltration/aggregation in lung tissue, there were significant differences between the groups (*p* = 0.006). Neutrophil infiltration/aggregation was significantly higher in the T/D group compared to groups C and CeO_2_ (*p* = 0.001 and *p* = 0.004, respectively) and was lower in group CeO_2_-T/D compared to group T/D (*p* = 0.013). The alveolar wall thickness was significantly higher in group T/D group than in group C and CeO_2_ (*p* < 0.001 and *p* = 0.001, respectively). Also, TLISs were significantly higher in group T/D compared to groups C and CeO_2_ (*p* < 0.001 and *p* = 0.002, respectively). TLISs were significantly lower in group CeO_2_-T/D compared to group T/D (*p* = 0.033) (Table 2 and Figures 5–8).

**Figure 9:**
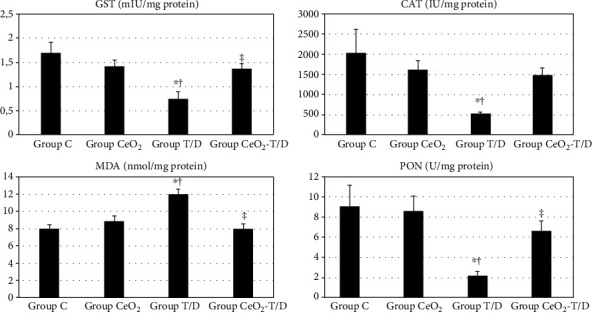
Lung tissue oxidative stress parameters. GST: glutathione S-transferase; CAT: catalase; MDA: malondialdehyde; PON: paraoxonase. ^∗^*p* < 0.05: compared to group C. ^†^*p* < 0.05: compared to group CeO_2_. ^‡^*p* < 0.05: compared to group T/D.

There were significant differences between groups when compared in terms of lung tissue GST, CAT, and PON enzyme activities and MDA levels (*p* = 0.002, *p* = 0.024, *p* = 0.008, and *p* < 0.001). GST enzyme activity was significantly lower in group T/D compared to groups C and CeO_2_ (0.74 ± 0.14 vs. 1.70 ± 0.22 IU/mg protein, *p* < 0.001, and 0.74 ± 0.14 vs. 1.42 ± 0.13 IU/mg protein, *p* = 0.004, respectively). It was significantly higher in group CeO2-T/D when compared to group T/D (1.37 ± 0.10 vs. 0.74 ± 0.14 IU/mg protein, *p* = 0.007). CAT enzyme activity was significantly lower in group T/D compared to groups C and CeO_2_ (526.40 ± 37.65 vs. 2038.02 ± 575.14 IU/mg protein, *p* = 0.004, and 526.40 ± 37.65 vs. 1610.35 ± 229.98 IU/mg protein, *p* = 0.028, respectively). Groups CeO_2_-T/D and C were similar in terms of CAT enzyme activity (1476.70 ± 184.00 vs. 2038.02 ± 575.14 IU/mg protein, *p* = 0.051). MDA levels were significantly lower in group T/D compared to groups C and CeO_2_ (11.97 ± 0.62 vs. 7.97 ± 0.49 nmol/mg protein, *p* < 0.001, and 11.97 ± 0.62 vs. 8.85 ± 0.63 nmol/mg protein, *p* = 0.001, respectively). MDA levels were also significantly lower in group CeO_2_-T/D when compared to group T/D (7.98 ± 0.58 vs. 11.97 ± 0.62 nmol/mg protein, *p* < 0.001). PON enzyme activity was significantly lower in group T/D compared to groups C and CeO_2_ (2.18 ± 0.43 vs. 9.09 ± 2.10 IU/mg protein, *p* = 0.002 and 2.18 ± 0.43 vs. 8.60 ± 1.46 IU/mg protein, *p* = 0.004, respectively). It was significantly higher in group CeO_2_-T/D when compared to group T/D (6.62 ± 1.00 vs. 2.18 ± 0.43 IU/mg protein, *p* = 0.034) (Figure 9).

**Figure 10:**
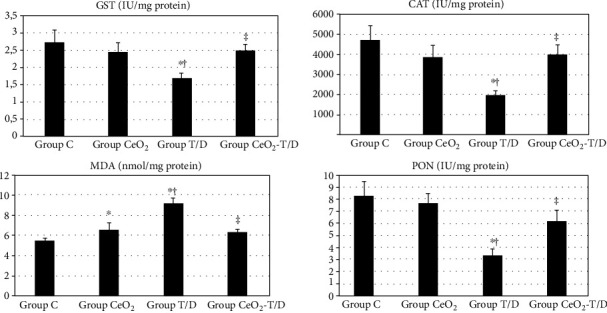
Renal tissue oxidative stress parameters. ^∗^*p* < 0.05: compared to group C. ^†^*p* < 0.05: compared to group CeO_2_. ^‡^*p* < 0.05: compared to group T/D.

Renal tissue GST, CAT, and PON enzyme activities and MDA levels were significantly different between groups (*p* = 0.049, *p* = 0.012, *p* = 0.004, and *p* < 0.001, respevtively). GST enzyme activity was significantly lower in group T/D compared to groups C and CeO_2_ (1.70 ± 0.14 vs. 2.73 ± 0.36 IU/mg protein, *p* = 0.009, and 1.70 ± 0.14 vs. 2.46 ± 0.27 IU/mg protein, *p* = 0.047, respectively). It was significantly higher in group CeO_2_-T/D when compared to group T/D (2.50 ± 0.18 vs. 1.70 ± 0.14 IU/mg protein, *p* = 0.037). CAT enzyme activity was significantly lower in group T/D compared to groups C and CeO_2_ (1976.54 ± 237.91 vs. 4715.22 ± 701.68 IU/mg protein, *p* = 0.002, and 1976.54 ± 237.91 vs. 3861.30 ± 588.71 IU/mg protein, *p* = 0.022, respectively). It was significantly higher in group CeO_2_-T/D when compared to group T/D (3992.94 ± 503.78 vs. 1976.54 ± 237.91 IU/mg protein, *p* = 0.015). MDA levels were significantly higher in group T/D compared to groups C and CeO_2_ (9.21 ± 0.47 vs. 5.44 ± 0.24 nmol/mg protein, *p* < 0.001, and 9.21 ± 0.47 vs. 6.53 ± 0.73 nmol/mg protein, *p* = 0.001, respectively). It was significantly lower in group CeO_2_-T/D when compared to group T/D (6.35 ± 0.19 vs. 9.21 ± 0.47 nmol/mg protein, *p* < 0.001). PON enzyme activity was significantly lower in group T/D compared to groups C and CeO_2_ (3.36 ± 0.49 vs. 8.28 ± 1.19 IU/mg protein, *p* = 0.001, and 3.36 ± 0.49 vs. 7.68 ± 0.82 IU/mg protein, *p* = 0.003, respectively). It was significantly higher in group CeO_2_-T/D when compared to group T/D (6.19 ± 0.91 vs. 3.36 ± 0.49 IU/mg protein, *p* = 0.036) (Figure 10).

## 4. Discussion

In this study, we evaluated the effects of CeO_2_ on lung and renal tissue following testicular T/D in rats. While there are few data on the effects of CeO_2_ in testicular I/R injury in the literature, the effects of CeO_2_ on distant organ injury in this I/R model are unknown. In order to investigate the effects of CeO_2_ in distant organ damage, we examined both histopathological and oxidative damage parameters in the lung and kidney in I/R injury. At the end of our study, it was shown that the use of CeO_2_ in the testicular T/D model significantly reduced both histopathological damage and oxidative damage in the lung and kidney.

Oxidative stress is a pathological process that occurs with the disruption of the balance between the production and accumulation of reactive oxygen radicals in cells and tissues [33]. Reactive oxygen radicals are the most important free radicals formed from oxygen in biological systems. ROS create lipid peroxidation by acting on fatty acids in the cell membrane. Lipid peroxidation causes cell apoptosis and necrosis. MDA is one of the secondary products formed during lipid peroxidation and is the most commonly used oxidative stress marker. Oxidative stress due to increased ROS during ischemia-reperfusion injury causes changes in mitochondrial oxidative phosphorylation, ATP depletion, increase in intracellular calcium, and increase in proteases and phosphatases [34]. Among the mechanisms against this oxidative damage, glutathione S-transferase plays an important role. It is responsible for the inactivation of electrophilic components and toxic substrates [35]. Catalase and paraoxonase are antioxidant enzymes. Paraoxonase is an ester hydrolase enzyme. It is named paraoxonase because it can hydrolyze paraoxon, the toxic metabolite of parathion, an organophosphate pesticide. In recent years, it has gained popularity due to its possible antioxidant effects [36]. Catalase is one of the first antioxidant defense mechanisms in the cell. It provides detoxification of hydrogen peroxide, which passes from mitochondria to the cytosol during oxidative damage [37].

Ischemia-reperfusion injury in the testicular tissue is the main pathophysiology of testicular torsion/detorsion. This damage causes an inflammatory response that includes an increase in reactive oxygen radicals and cytokines. In addition, testicular torsion/detorsion causes an increase in oxidative stress markers and a decrease in antioxidant enzyme levels [38, 39]. In our study, we investigated the effects of testicular torsion and cerium oxide on oxidative stress by measuring MDA, a marker of oxidative stress, and the activities of GST, CAT, and PON against oxidative damage. Shokoohi et al., in their study in rats, showed that testicular T/D increased lipid peroxidation, thus increasing serum MDA levels [40]. Similarly, in the study of Moghimian et al., it was shown that testicular T/D increased MDA levels and decreased MDA levels with the administration of antioxidant vitamin C [5]. The results of our study were also compatible with the results of these studies. It has been shown that MDA levels increase in lung and renal tissues with testicular T/D and decrease with the administration of cerium oxide.

The effects of testicular T/D have been shown by many studies. In the study of Moghimian et al., in which they investigated the effects of Syzygium aromaticum in a rat testis T/D model, it was shown that systemic antioxidant enzyme levels were decreased (superoxide dismutase and glutathione peroxidase) in T/D, and enzyme levels were improved after Syzygium aromaticum was given [1]. In the study by Ameli et al., in which they investigated the effects of tadalafil and verapamil on oxidative stress in rat testis T/D, it was shown that the levels of systemic antioxidant enzymes were decreased (superoxide dismutase and glutathione peroxidase), and the levels were increased with tadalafil and verapamil [2]. In the testicular T/D model performed by Yuvanc et al. in rats, it was shown that PON levels decreased and following antioxidant administration PON levels increased [41]. In these studies, serum antioxidant levels were examined. In our study, we evaluated the antioxidant enzyme levels in lung and kidney tissues. As a result, we showed that antioxidant enzyme levels were low in testis T/D in lung and kidney tissues, but antioxidant enzyme levels increased in tissues with cerium oxide administration despite T/D. In the study of Akdemir and Tanyeli, oxidative damage and antioxidant enzyme levels in the testis and lung tissues were evaluated after testicular T/D. Similar to our study, it has been shown that oxidative damage in the lung tissue increased and antioxidant enzyme levels were low after testicular T/D [42]. Ischemia-reperfusion injury causes damage to distant organs as well as local damage [43]. There is information in the literature that testicular T/D is associated with lung injury [42]. However, apart from this study, we could not find any other publication in the literature showing the relationship between testicular T/D and distant organ damage. In our study, we demonstrated that after testicular ischemia-reperfusion injury, ischemia-reperfusion injury occurs in both lung and kidney tissues.

Studies have shown that the duration and degree of testicular torsion are related to the severity of ischemic injury [40, 44]. Turner et al. showed that spermatogenesis was permanently impaired within 1 hour of testicular torsion of 720° in rats, and germ cell apoptosis, neutrophil infiltration, and oxidative damage occurred after detorsion. Therefore, they stated that testicular T/D can be considered as a typical I/R injury [45]. The prognosis of testicular torsion is related to the duration of the torsion, and the resulting oxidative damage leads to different degrees of damage. Bilommi et al. studied the efficacy of glutathione in testicular T/D using a 3-hour reperfusion model after 4 hours of ischemia [14]. In the study of Arya et al., 3 hours of ischemia followed by 3 hours of reperfusion was established [46]. In both studies, injury in the testicular tissue was examined and it was reported that the testicular tissue injury was revealed in the testicular torsion/detorsion models they used. In studies where shorter ischemia-reperfusion times were used, it was also shown that oxidative damage to the testicles and lungs occurred in a 2-hour reperfusion model after 2-hour ischemia used in studies in which Hirst and colleagues studied the effects of myricetin on rat testicular torsion/detorsion [47]. In the study in which Abbasoğlu and colleagues looked at the effects of taurine and carnosine on testicular torsion/detorsion in rats, ischemia and reperfusion times were determined as 2 hours each. As a result, it has been shown that histopathological and oxidative damage to testicular tissue occurred with these periods [9]. We used the 120-minute ischemia and then the 120-minute reperfusion model for the testicular torsion/detorsion model. However, we did not study the damage on testicular tissue. However, the histopathological changes and the changes in the oxidative stress parameters of the lung and kidneys have revealed that the model we used caused I/R damage in distant organs.

The reduction of oxidative damage caused by ischemia-reperfusion injury has become the target of many drug treatments. Cerium oxide is a nanoparticle that has been shown to be useful for use in medical and nonmedical fields [48, 49]. Cerium oxide exists in nature in two basic forms; cerium IV oxide (CeO_2_) and cerium III oxide (Ce_2_O_3_). CeO_2_ exists in a more stable phase at room temperature and atmospheric conditions. CeO_2_ nanoparticles are known to exist in two oxidative states; +4 and +3 oxidation states. This dual oxidation state of the nanoparticle means that these nanoparticles have oxygen vacancies. This dual oxidative state enables the emergence of antioxidant properties [18, 50, 51]. Its antioxidant properties have been proven by many different models of I/R injuries. Among these, lower extremity, hepatic myocardial, intestinal, and spinal cord I/R injuries are involved [15–19, 23, 52, 53]. The effects of cerium oxide on testicular I/R injury are examined in studies. In these studies, cerium oxide acted as an antioxidant and reduced oxidative and histopathological damage to testicular tissue after reperfusion [46–54]. However, in our study, the effects of cerium oxide on testicular tissue were not examined and it was shown to reduce histopathological and oxidative damage in the lung and kidney tissue.

While there are many experimental animal studies and cell culture studies on cerium oxide today, there is no clinical use of this nanoparticle yet. These studies provide information about the use of cerium oxide for diagnosis and treatment in different pathophysiological conditions [50–55]. In this direction, as in other studies, we hope that the result of our study may be a study that can shed light on other experimental studies and clinical use.

The limitations of our study are that it will not be possible to adapt it to the clinical era since it is an animal study. Although the rat testicle has differences from the human testicle, rats are often used for testicular T/D models. This is due to the fact that lesions in the testicular torsion studies are comparable to those in the human testicles. Another limitation is that the experiment was performed without power analysis. The reason we did not perform a power analysis is because the ethics committee considers these numbers appropriate for the welfare of animals. We have reported the results of the study with 0.5 mg/kg ip dose we used for cerium oxide. The fact that we did not study the changes in the degree of oxidative damage at different doses may be another limitation of the study.

As a result of our study, it was shown that cerium oxide can reduce the oxidative and histopathological injury in the lung and kidney after testicular ischemia reperfusion. Reducing the oxidative damage associated with I/R has become the goal of drug studies conducted in this area. In many studies, many molecules that can inhibit oxidative stress have also been studied. It may be thought that cerium oxide may also be one of these potential inhibitors in testicular I/R injury. With this result, our study is the first to investigate the effects of cerium oxide on distant organ damage in a testicular I/R model. For this reason, considering the limitations of the study, contributions to the literature can be made by supporting the results with future studies to be carried out with new methods.

## Figures and Tables

**Table 1:** Histopathological findings in renal tissue (mean ± SE).

	Group C (*n* = 6)	Group CeO_2_ (*n* = 6)	Group T/D (*n* = 6)	Group CeO_2_-T/D (*n* = 6)	*p* ^∗∗^
Glomerular vacuolization (GV)	0.17 ± 0.17	0.33 ± 0.21	1.33 ± 0.33^∗^^†^	0.67 ± 0.21^‡^	0.012
Tubular dilatation (TD)	0.33 ± 0.21	0.50 ± 0.22	1.17 ± 0.17^∗^^†^	0.67 ± 0.21	0.049
Vascular vacuolization and hypertrophy (VVH)	0.33 ± 0.21	0.50 ± 0.22	1.00 ± 0.00	0.50 ± 0.22	0.107
Tubular cell degeneration and necrosis (TCDN)	0.17 ± 0.17	0.33 ± 0.21	1.33 ± 0.21^∗^^†^	0.67 ± 0.21^‡^	0.003
Bowman space dilatation (BSD)	0.33 ± 0.21	0.33 ± 0.21	0.83 ± 0.31	0.50 ± 0.22	0.434
Tubular hyaline cylinder (THC)	0.50 ± 0.22	0.33 ± 0.21	1.00 ± 0.00	0.83 ± 0.17	0.053
Lymphocyte infiltration (LI)	0.33 ± 0.21	0.33 ± 0.21	1.33 ± 0.21^∗^^†^	0.83 ± 0.31	0.046
Tubular cell spillage (TCS)	0.33 ± 0.21	0.50 ± 0.22	1.17 ± 0.17^∗^^†^	0.67 ± 0.21	0.049

*p* ^∗∗^: significance level with the Kruskal-Wallis test *p* < 0.05. ^∗^*p* < 0.05: compared to group C. ^†^*p* < 0.05: compared to group CeO_2_. ^‡^*p* < 0.05: compared to group T/D.

**Table 2:** Histopathological findings in lung tissue (mean ± SE).

	Group C (*n* = 6)	Group CeO_2_ (*n* = 6)	Group T/D (*n* = 6)	Group CeO_2_-T/D (*n* = 6)	*p* ^∗∗^
Neutrophil infiltration/aggregation	0.33 ± 0.21	0.50 ± 0.22	1.50 ± 0.22^∗^^†^	0.67 ± 0.21^‡^	0.006
Alveolar wall thickening	0.33 ± 0.21	0.50 ± 0.22	1.50 ± 0.22^∗^^†^	1.00 ± 0.00^∗^	0.001
Total lung injury score	0.67 ± 0.42	1.00 ± 0.44	2.83 ± 0.30^∗^^†^	1.67 ± 0.21^∗^^‡^	0.002

*p* ^∗∗^: significance level with the Kruskal-Wallis test *p* < 0.05. ^∗^*p* < 0.05: compared to group C. ^†^*p* < 0.05: compared to group CeO_2_. ^‡^*p* < 0.05: compared to group T/D.

## Data Availability

The data that support the findings of this study are available from the corresponding author (Arslan, M), upon reasonable request (Mustafa Arslan mustarslan@gmail.com).
